# Deep UV-excited fluorescence microscopy installed with CycleGAN-assisted image translation enhances precise detection of lymph node metastasis towards rapid intraoperative diagnosis

**DOI:** 10.1038/s41598-023-48319-7

**Published:** 2023-12-04

**Authors:** Junya Sato, Tatsuya Matsumoto, Ryuta Nakao, Hideo Tanaka, Hajime Nagahara, Hirohiko Niioka, Tetsuro Takamatsu

**Affiliations:** 1 Graduate School of Information Science and Technology, Osaka University, 1-5, Yamadaoka, Suita, Osaka 565-0871 Japan; 2 Department of Pathology and Cell Regulation, Kyoto Prefectural University of Medicine, 465 Kajiicho, Kawaramachi-Hirokoji, Kamigyo-ku, Kyoto, 602-8566 Japan; 3 Institute for Datability Science, Osaka University, 2-8 Yamadaoka, Suita, 565-0871 Japan; 4 Department of Radiology, Osaka University Graduate School of Medicine, 2-2, Yamadaoka, Suita, Osaka 565-0871 Japan; 5 Department of Artificial Intelligence Diagnostic Radiology, Osaka University Graduate School of Medicine, 2-2, Yamadaoka, Suita, Osaka 565-0871 Japan; 6 Department of Medical Photonics, Kyoto Prefectural University of Medicine, 465 Kajiicho, Kawaramachi-Hirokoji, Kamigyo-ku, Kyoto, 602-8566 Japan

**Keywords:** Biophotonics, Machine learning, Microscopy, Cancer

## Abstract

Rapid and precise intraoperative diagnosing systems are required for improving surgical outcomes and patient prognosis. Because of the poor quality and time-intensive process of the prevalent frozen section procedure, various intraoperative diagnostic imaging systems have been explored. Microscopy with ultraviolet surface excitation (MUSE) is an inexpensive, maintenance-free, and rapid imaging technique that yields images like thin-sectioned samples without sectioning. However, pathologists find it nearly impossible to assign diagnostic labels to MUSE images of unfixed specimens; thus, AI for intraoperative diagnosis cannot be trained in a supervised learning manner. In this study, we propose a deep-learning pipeline model for lymph node metastasis detection, in which CycleGAN translate MUSE images of unfixed lymph nodes to formalin-fixed paraffin-embedded (FFPE) sample, and diagnostic prediction is performed using deep convolutional neural network trained on FFPE sample images. Our pipeline yielded an average accuracy of 84.6% when using each of the three deep convolutional neural networks, which is a 18.3% increase over the classification-only model without CycleGAN. The modality translation to FFPE sample images using CycleGAN can be applied to various intraoperative diagnostic imaging systems and eliminate the difficulty for pathologists in labeling new modality images in clinical sites. We anticipate our pipeline to be a starting point for accurate rapid intraoperative diagnostic systems for new imaging modalities, leading to healthcare quality improvement.

## Introduction

The number of patients with cancer is increasing with global population growth and ageing, and a total of 19.3 million patients were newly diagnosed in 2020^1^. The gold standard treatment for radical cure of cancer is surgery, and detailed surgical procedures are determined according to the tumour size and metastasis to surrounding lymph nodes.

Rapid intraoperative diagnosis plays an important role in detecting the presence of sentinel lymph node metastases for some malignancies (e.g., gastric and breast cancers). It is a direct decision-making aid for surgeons that affects surgical progress and patient prognosis. Although pathological diagnosis based on tissue sections and staining is the gold standard for cancer diagnosis, these procedures take several days to complete^2^. For rapid intraoperative diagnosis of lymph node metastases, a frozen section procedure is widely used; however, this process is associated with some problems, such as sample degradation due to the formation of ice crystals and the requirement of experience in handling the samples. Recently, the OSNA™ (One-Step Nucleic Acid Amplification) method has been developed, enabling the determination of metastasis based on the amplification and detection of cytokeratin 19 mRNA contained in the solubilised lymph nodes. This method has been mainly applied to the sentinel lymph node of breast cancer, and highly accurate results have been obtained without pathological procedures^3^. However, these two methods, namely frozen section and OSNA, generally require approximately 20 min for the entire detection process. Another approach is the highly sensitive and specific 5-aminolevulinic acid (5-ALA)-induced rapid fluorescence imaging method^4,5^. We have previously developed a device that automatically detects lymph node metastasis by quantitatively evaluating the 5-ALA-induced protoporphyrin IX (PpIX) fluorescence intensity while eliminating tissue autofluorescence. However, the activity of the ATP-binding cassette transporter G2 is very high in breast cancer cells, making 5-ALA unsuitable for detecting breast cancer lymph node metastasis^6^.

Microscopy with ultraviolet surface excitation (MUSE) is a less expensive and maintenance-free imaging system with the potential for rapid intraoperative diagnosis^7^. As deep-ultraviolet (DUV) light in the 250–300 nm wavelength range is easily absorbed and scattered by biological samples, resulting in excitation only near the sample surface, MUSE produces images like thin-sliced sample sections without sectioning. Observing cross-sections of the excised tissue is adequate for the pathologist to distinguish clearly between the nucleoplasm, nucleolus, and cytoplasm. Our group has reported a staining method for MUSE using Terbium ion and Hoechst/DAPI, which provided clear contrast images and required only 5 min of staining time^7^.

In our previous study, formalin-fixed paraffin-embedded (FFPE) sections of lymph nodes from gastric cancer patients stored in a hospital were stained and observed with MUSE to create a fluorescent image dataset^8^. These images were utilised for training a deep convolutional neural network (DCNN), achieving high diagnostic accuracy comparable to that of a pathologist using HE images. The advantage of MUSE is that metastatic lesions can be detected in a short time using unfixed specimen images instead of FFPE images. The contrast of an unfixed specimen image differs from that of an FFPE image, such as that at the cell boundary. Thus, if the unfixed specimen image of the test data can be translated to an FFPE-like image, a high detection rate can be achieved. FFPE images allow us to train image classification models, such as DCNN, because supervised labels can be assigned to cancerous and other regions by referring to the serially sectioned HE images. As labelling is not possible for MUSE images of unfixed specimens, image classification models are not trainable in a supervised manner.

In this study, we propose a rapid diagnostic pipeline model that combines a CycleGAN^9^ to translate MUSE images of unfixed lymph nodes to FFPE sample images with a DCNN trained on FFPE sample images for diagnostic prediction. CycleGAN is a successful image translation method that uses unpaired image data. Some CycleGAN-based methods have been reported to correct the colour of HE images at different facilities^10–12^, translate the images to a different staining method^13–16^, and improve accuracy by augmentation^17^. First, we trained an image translation model that translates between unfixed and FFPE MUSE images using CycleGAN and a diagnostic prediction DCNN model using FFPE sample images. Next, the CycleGAN and DCNN models were coupled to form a pipeline model, and we compared the diagnostic prediction accuracy based on MUSE images of unfixed lymph nodes with and without CycleGAN.

## Methods

### Clinical specimens

All clinical experiments were conducted with the approval of the Ethics Committees of the Kyoto Prefectural University of Medicine (approval no.: ERB-C-1038) as well as in accordance with the guidelines from the committees and regional laws related to clinical research. The lymph nodes used in this study were obtained from patients during gastric cancer surgery at the University Hospital, Kyoto Prefectural University of Medicine. Informed consent was obtained from all participants. Patients were diagnosed as metastasis-positive or metastasis-negative based on postoperative pathological examination. None of the patients had received preoperative radiotherapy or chemotherapy. The number of prepared FFPE MUSE images for metastasis-positive and metastasis-negative samples was 38 and 18, respectively. These MUSE images were identical to those used in our previous study^8^. They were chosen such that they included all the common histological types of gastric carcinoma. For developing the CycleGAN translation model, the FFPE lymph nodes mentioned above and 19 metastasis-positive and 9 metastasis-negative unfixed lymph nodes obtained from 28 patients were used. For the MUSE images of unfixed lymph nodes, we had 45 metastasis-positive and 46 metastasis-negative images.

### Staining protocol and fluorescence microscopy

Complete details of the sample preparation and imaging methods have been described in a previous study by Kumamoto et al.^7^. Briefly, surgically obtained lymph nodes were sliced and immersed in 95% or 99.5% ethanol, rinsed with HEPES buffer (10 mM HEPES, pH 7 adjusted with NaOH), immersed in 100% D_2_O HEPES buffer solution containing TbCl_3_ (TBH03XB, Kojundo Chemical Laboratory, Saitama, Japan) and Hoechst 33342 (Dojindo Molecular Technologies), and then rinsed with 100% D_2_O HEPES buffer. Stained specimens were then set on an inverted microscope (IX71, Olympus) equipped with an objective lens (UPLFLN 10×, Olympus). The specimen’s surface was illuminated with a DUV light (30 mW and 5 mm in power and diameter, respectively) emitting from an LED (M285L5, Thorlabs). Fluorescence emitted from the specimen was collimated with the objective lens and imaged using a CMOS camera (UI-3180CP-C-HQ Rev. 2, OnSemi). An optical filter (FF01-464/547-25, Semrock) was set between the objective and imaging lenses to attenuate DUV light. A schematic representation of the image acquisition protocol is shown in Fig. S1.

FFPE sections were obtained after standard formalin fixation and paraffin embedding, followed by thin sectioning at 4 µm. Before the MUSE imaging, the FFPE sections were deparaffinised and stained using the same method as that used for unfixed lymph nodes.

### Training of CycleGAN

CycleGAN is a model with two generators and two discriminators that learns to translate between the domains of the two image datasets. The generator outputs an image in the other domain from the input image data domain, and the discriminator predicts which domain the output image belongs to. Two pairs of generator-discriminator perform domain translations and learn bi-directional translations. By training these pair models simultaneously, CycleGAN can output images that are restyled from one to the other. Each of the MUSE images of unfixed lymph nodes was divided into small patches composed of 256 × 256 pixels. The training dataset consisted of 256 × 256 pixels size images cropped from all the MUSE images of unfixed lymph nodes data used in this study. In this process, the 256 × 256 pixels size cropping window was scanned vertically and horizontally in 10 pixels steps. Thus, 1,364,540 patch images were prepared. The MUSE image dataset of the FFPE samples was prepared using the patch-image dataset from our previous study^8^. The original patch-image size was 278 × 278. Each patch was then resized into 256 × 256 pixels, and pixel values were scaled from − 1 to 1 by dividing by 127.5 and subtracting 1 for normalization in the CycleGAN model.

The loss of CycleGAN consists of adversarial loss *l*_adv_, cycle consistency loss *l*_cycle_, and identity loss *l*_identity_. First, adversarial loss, which was introduced by Goodfellow et al.^18^, was set up so that the generator *G* and discriminator *D* were simultaneously trained by trying to defeat each other. Given the images in domain A (*X*_A_^real^), those in domain B (*X*_B_^real^), the generative model that transforms domain A into domain B (*G*_AB_), and the model that discriminates whether *X*_B_ is true or false (*D*_B_), adversarial loss in domain B *l*_*adv*_^B^ was expressed as:

$$\begin{array}{c}{l}_{adv}^{B}={E}_{{X}_{B}}\left[{logD}_{B}{\left({X}_{B}^{real }\right)}^{2}\right]+{E}_{{X}_{A}}\left[{\left({logD}_{B}\left({G}_{AB}\left({X}_{A}^{real }\right)\right)-1\right)}^{2}\right]\\ \end{array},$$

where E_X_ is the expected value over all real data instances. *l*_*adv*_^A^ was also calculated in the same way using G_BA_ and D_A_. The total adversarial loss was the sum of *l*_*adv*_^A^ and *l*_*adv*_^B^. Next, cycle consistency loss ensures the reversibility of the translation and makes it closer to a one-to-one conversion. The loss *l*_cycle_ was expressed as follows:

$${{l}^{cycle}=E}_{{X}_{A}}\left[{\parallel {G}_{BA}\left({G}_{AB}\left({X}_{A}^{real}\right)\right)-{X}_{A}^{real}\parallel }_{1}\right]+{E}_{{X}_{B}}\left[{\parallel {G}_{AB}\left({G}_{BA}\left({X}_{B}^{real}\right)\right)-{X}_{B}^{real}\parallel }_{1}\right],$$

where ‖·‖_1_ is L1-norm. The first term refers to cycle loss in domain A and the second term refers to cycle loss in domain B. Third, the identity loss regularises a colour change and may ensure common elements within a domain as proposed by Taigman et al.^19^. The loss *l*_identity_ was expressed as:

$${l}^{identity}={E}_{{X}_{A}}\left[{\parallel {G}_{BA}({X}_{A}^{real})-{X}_{A}^{real}\parallel }_{1}\right]+{E}_{{X}_{B}}\left[{\parallel {G}_{AB}\left({X}_{B}^{real}\right)-{X}_{B}^{real}\parallel }_{1}\right].$$

Using the above three types of loss, the total loss was

$${l}^{total}={l}^{adv}+{\alpha l}^{cycle}+{\beta l}^{identity},$$

where α and β are relative ratios; in this study, we set α = 10 and β = 1.

Adam was used as an optimiser, and the learning rate was set at 0.0002 based on the original study^9^. The model was trained on 48 epochs.

### Training of DCNN models

Three image classification DCNN models were adopted, including Inception v3^20^, DenseNet121^21^, and EfficientNetB4^22^. These models have been previously applied in medical image classification, achieving high accuracy of 98.4%, 99.1%^23^, and 94.9%^24^, respectively, in the colorectal cancer prediction task. We downloaded the weights pre-trained on ImageNet^25^ and performed transfer learning. In these models, global average pooling is preceded before the output layer composed of 2 nodes. In the transfer learning of these three models, only the weights of the last fully connected layer are trained first, and then the weights of the whole model are trained. As an optimiser, stochastic gradient descent was used for all models. All MUSE images of FFPE lymph nodes were divided into 27,704 small patches of 256 × 256 pixels in size without them overlapping each other. Of these, 10,502 metastasis-positive and 9931 metastasis-negative patches were used for training, 1988 metastasis-positive and 1455 metastasis-negative patches were used for validation, and 1834 metastasis-positive and 1994 metastasis-negative patches were used for testing. During data partitioning, the patch images were treated together as a group for each patient and not as split. Each image patch was normalized to a range between 0 and 1 by dividing each pixel value by the maximum pixel value of the image before being input into the model. In all DCNN models, training was performed with the training dataset, and the weights were saved when the accuracy for the validation dataset was the highest. Saved weights were loaded during the evaluation of the performance of each DCNN model using the test dataset.

### Sliding window mapping

The mapping data was constructed using a sliding window process. The window size was 256 × 256 pixels square with 10 pixels steps, inputting the patch-image cropped with the window size into the CycleGAN and DCNN pipeline model and sequentially obtaining the classified results. The window scanned the entire image, and the mapping data were output. To prevent the disappearance of the metastasis-positive region present at the peripheral regions of the image during the later majority processing steps, mirroring of 128 pixels was performed at the top, bottom, left, and right of the original pathological image before performing the mapping process. The size of the mapping data finally obtained by the above means was 259 × 204 pixels from the original MUSE image (2592 × 2048) obtained with a 10× objective lens.

### Majority voting process

The majority voting process was applied to the image after mapping. Of note, mapping by the deep-learning pipeline is a separate process from the majority voting process. If the mapping data were created with a sliding window of 10 pixels steps, a sufficiently small metastasis-positive area in the fluorescence pathology image was converted to a size of about 26 pixels square because the window size was 256 × 256. Based on this size, a 26 × 26 pixels kernel was defined for majority voting, and a new positive/negative pixel was output when the ratio of the positive/negative pixels included in the kernel exceeded the threshold α (a detailed schematic image is shown in Fig. S2). The α was set to 70%, 80%, 90%, and 95%, respectively.

### Computer and software used for calculation

For training all models, a custom-made GPU server with a CPU (Xeon Gold 5218, 2.30 GHz, Intel) and a GPU (Quadro RTX 8000, 48 GB, NVIDIA) was used. For the mapping process, another GPU (A100, 80 GB, NVIDIA) was used. The installed OS was Ubuntu 16.04 LTS. We used TensorFlow version 2.2.0 and Keras version 2.4.3 to build the CycleGAN and DCNN pipeline model.

## Results

### MUSE images of unfixed lymph nodes from gastric cancer

**Figure 1:**
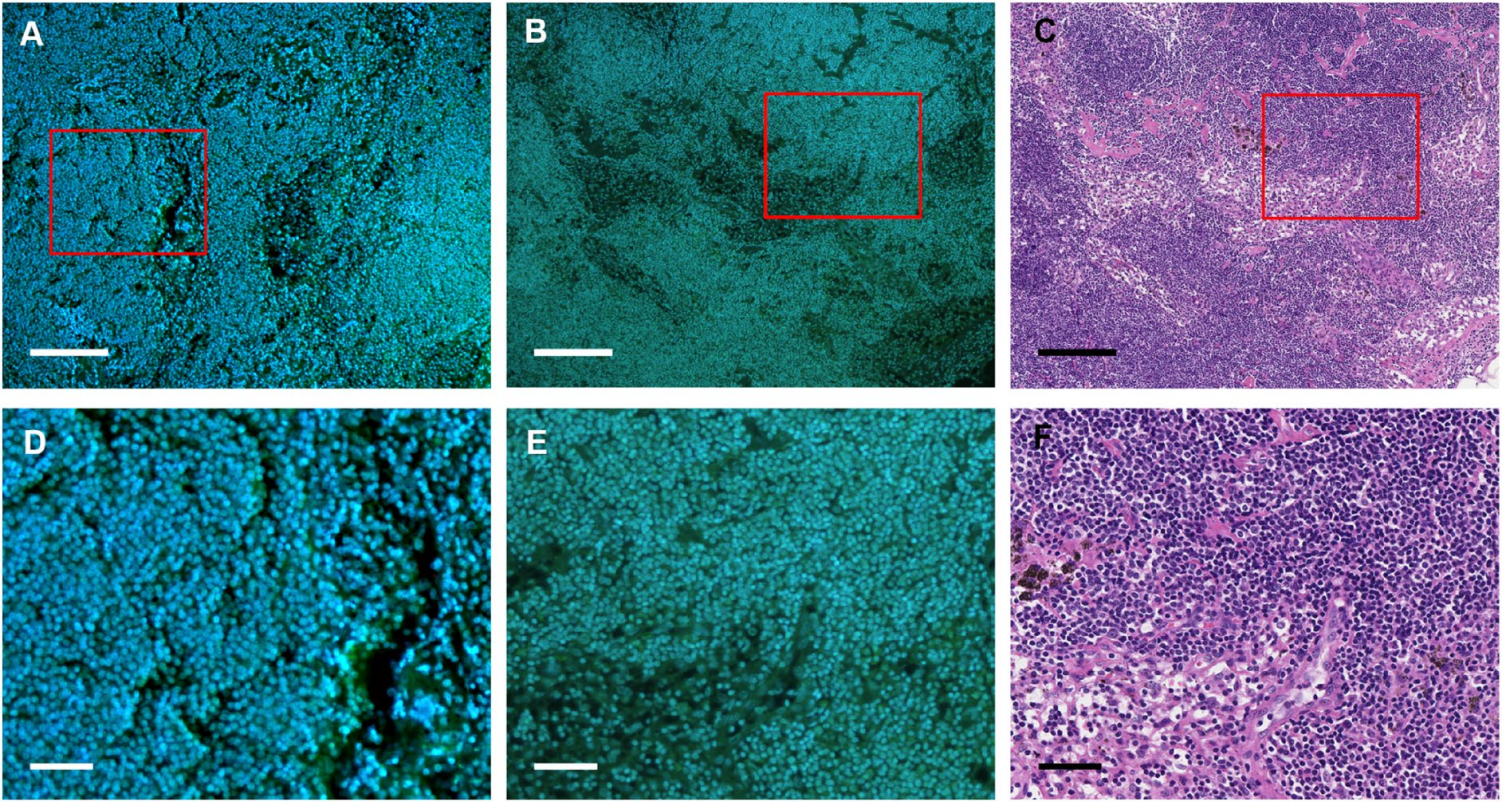
Representative MUSE and HE Images of metastasis-negative lymph nodes. (**A,D**) MUSE images of unfixed lymph nodes and (**B,E**) FFPE lymph nodes; (**C,F**) the corresponding HE images. All images were acquired using the lymph nodes obtained from patients with gastric cancer and were diagnosed as metastasis-negative. Zoomed-in versions of the red squared insets from each image (**A–C**) are presented below the source images (**D–F**). Scale bars: 200 µm (**A–C**) and 100 µm (**D–F**). *FFPE* formalin-fixed paraffin-embedded, *HE* haematoxylin and eosin staining, *MUSE* microscopy with ultraviolet surface excitation.

**Figure 2:**
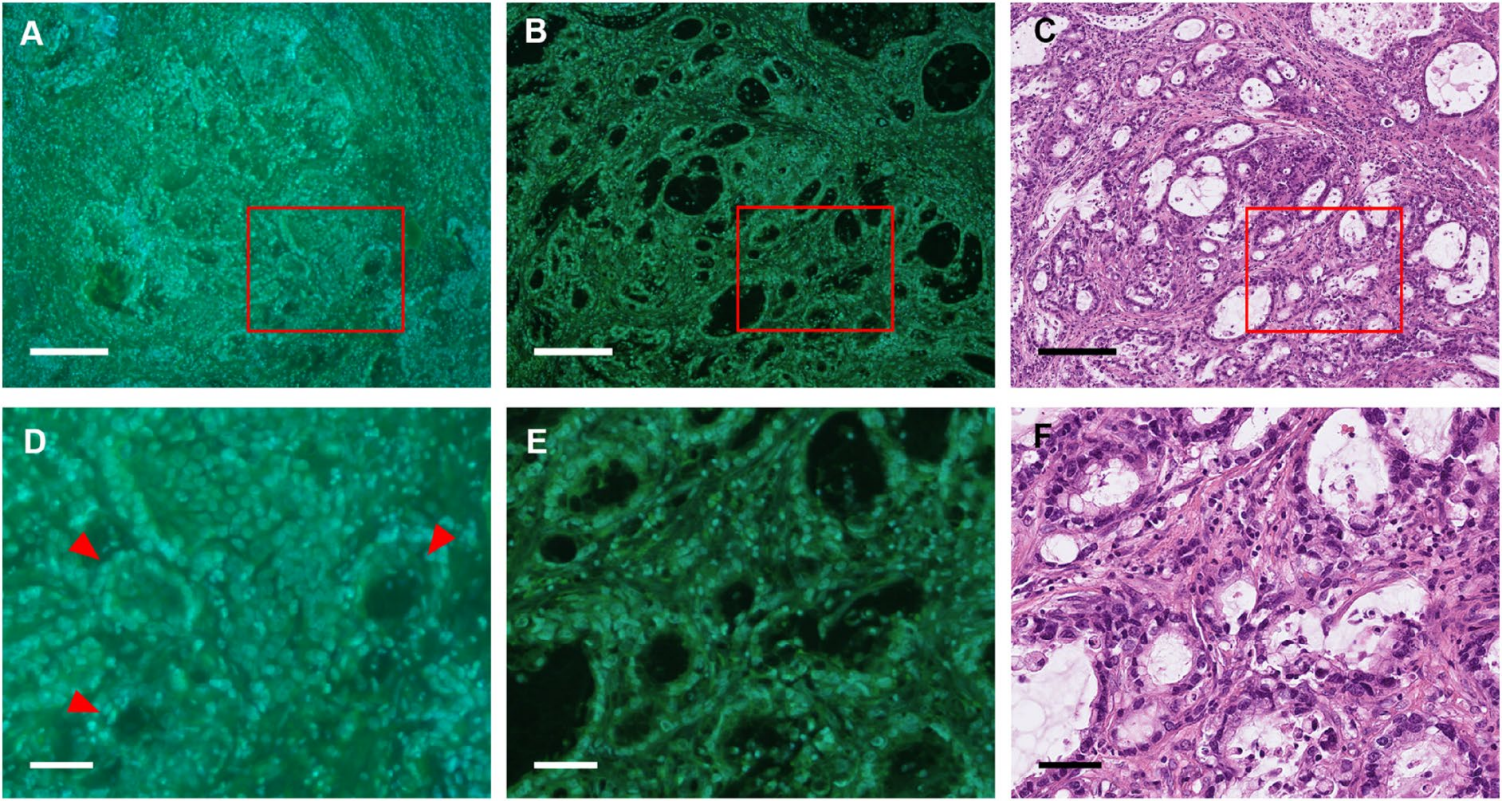
Representative MUSE and HE Images of metastasis-positive lymph nodes. (**A,D**) MUSE images of the unfixed lymph nodes and (**B,E**) FFPE lymph nodes; (**C,F**) the corresponding HE images. All images were acquired using the lymph nodes obtained from patients with gastric cancer and were diagnosed as metastasis-positive. Zoomed-in versions of the red squared insets from each image (**A–C**) are presented below the source images (**D–F**). Scale bars: 200 µm (**A–C**) and 100 µm (**D–F**). *FFPE* formalin-fixed paraffin-embedded, *HE* haematoxylin and eosin staining, *MUSE* microscopy with ultraviolet surface excitation.

We obtained MUSE images of unfixed lymph nodes from patients with gastric cancer using a previously reported protocol (described in “Methods”, Fig. S1). The MUSE images of unfixed specimens were obtained in the clinical setting approximately 5 min after the start of biopsy specimen processing without thin sectioning (Fig. S1). Figures 1 and 2 show representative MUSE images of metastasis-negative and metastasis-positive tissues obtained from unfixed lymph nodes, along with MUSE and HE images of FFPE specimens prepared from the same lymph nodes, respectively. The red squares in the images have been enlarged and displayed below each image. In the MUSE images from unfixed lymph nodes, gland-formed cancer tissues and normal lymphoid tissues were clearly visible, as in those in the HE images of FFPE specimens. Cancer tissues were identified by experienced pathologists in the HE images of FFPE specimens shown in Fig. 2C,F. Similarly, cancer glands were also identified in the metastasis-positive MUSE images of unfixed specimens (Fig. 2A,D). When comparing the MUSE images derived from unfixed and FFPE lymph nodes, the former images had higher fluorescence background originating from outside the focal plane due to the thicker specimen, resulting in a different contrast for these images. Additionally, the background intensity was not uniform in different image areas. Such background irregularities are thought to be caused by a complex combination of various factors, including cell density, the intensity of fluorescence of individual cells, and light scattering by cellular tissues. Cellular tissue structures also appeared to have shrunken in FFPE images due to the effects of fixation and dehydration employed during sample preparation. These differences in the appearance of the images make it difficult to diagnose the unfixed specimens using DCNN models trained on FFPE images. The issue of degraded performance of classification models due to differences in the appearance of cellular histology is also known to occur with inter-institutional variability in HE staining^11^.

### Image translation using CycleGAN

**Figure 3:**
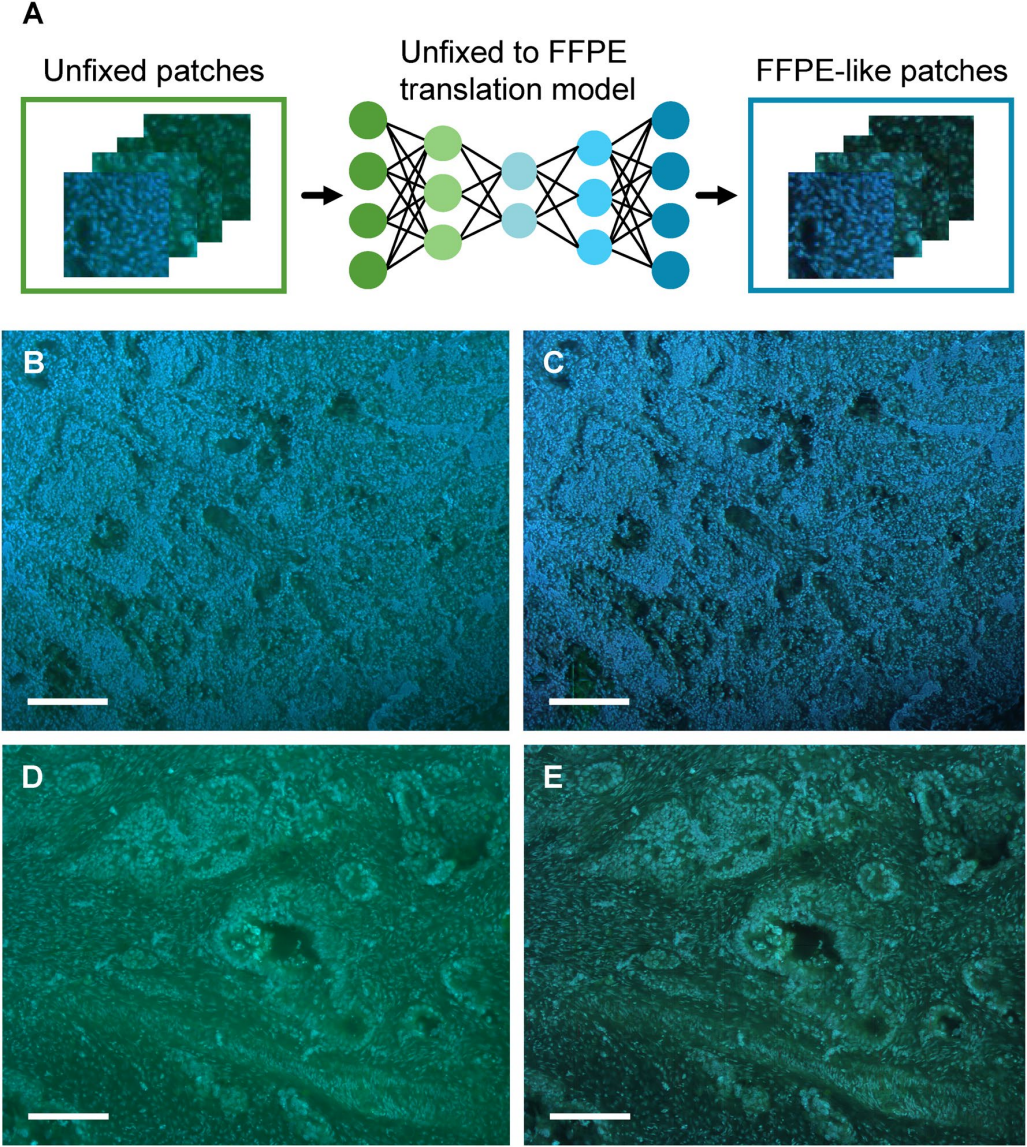
CycleGAN-assisted image translation. (**A**) Patch images extracted from the whole MUSE image patches are translated into FFPE-like via CycleGAN. (**B–E**) MUSE images before and after CycleGAN translation. (**B**) Unfixed metastasis-negative and (**D**) metastasis-positive images were translated into FFPE-like images (**C,E**). Scale bars: 200 µm. *FFPE* formalin-fixed paraffin-embedded, *MUSE* microscopy with ultraviolet surface excitation.

We adopted CycleGAN to translate between MUSE images of unfixed and FFPE lymph nodes, which allowed us to diagnose unfixed lymph nodes with the classification DCNN model trained using only FFPE images. CycleGAN trains on bi-directional image modality translations simultaneously. Figure 3A shows the MUSE images translated from the unfixed patch images to FFPE-like patch images using the trained CycleGAN. Original unfixed lymph node images had blurred edges of the nucleolus, cytoplasm, and other cell organelles, as well as a low contrast (Fig. 3B,D). Unwanted non-uniform background fluorescence was also observed. On the contrary, the images translated through CycleGAN had a clear contrast and corrected background compared to the original images (Fig. 3C,E). During image translation using CycleGAN, the images of the sizes shown in Fig. 3 (2592 × 2048) were too large to be translated all at once; hence, the images were cut into patches, translated sequentially, and then stitched together. The quality of the translation was high enough, such that the joints between the patched images were difficult to distinguish. For the training of CycleGAN, the images in Fig. 3B,D were divided into 256 × 256 patch-image sizes (see Details of CycleGAN training in “Methods” section).

### Training of DCNN models for cancer classification using FFPE MUSE images

**Figure 4:**
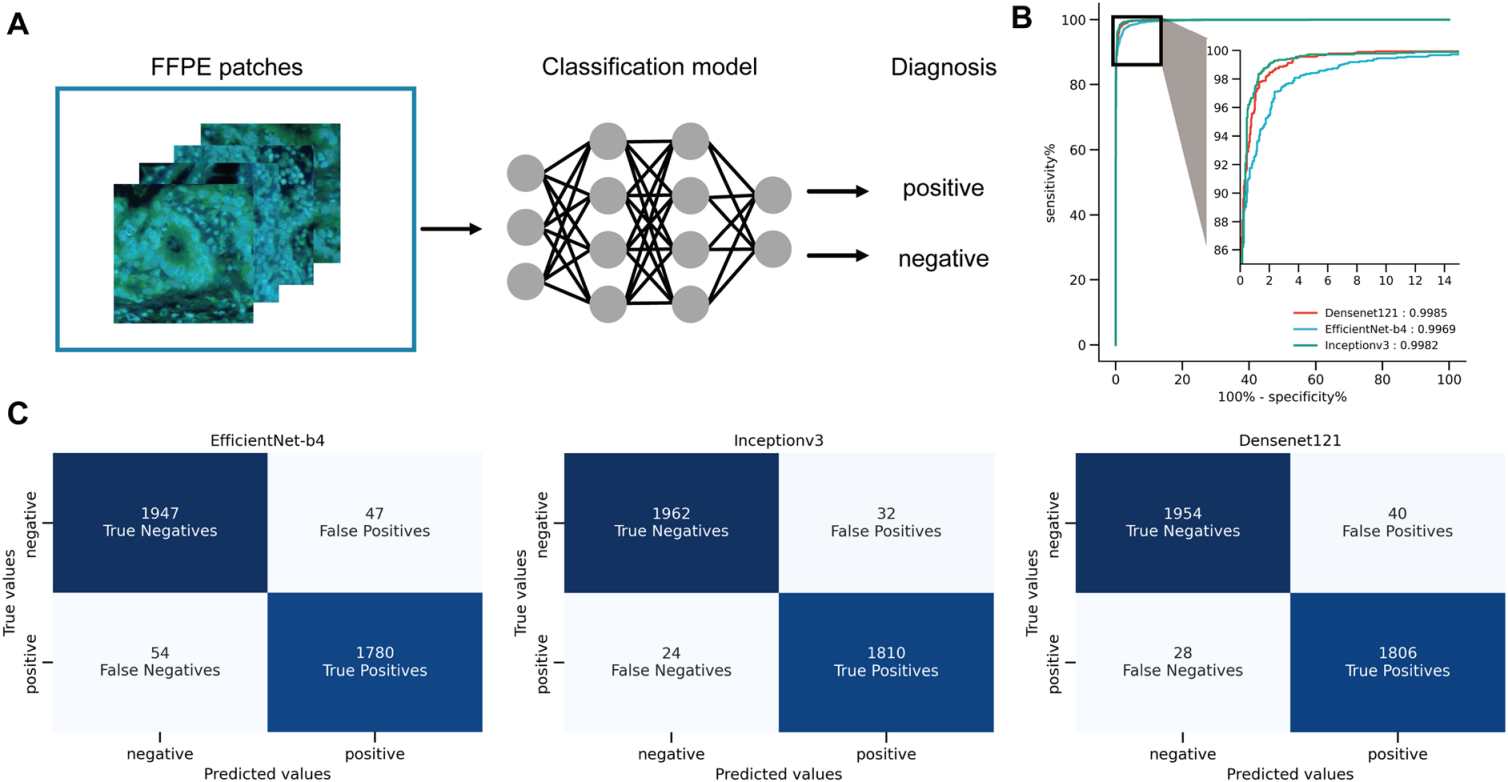
Performance of DCNN Classification Models on MUSE Image patches. (**A**) Conceptual diagram depicting DCNN model for image classification. The MUSE image patches of FFPE lymph nodes are the input, and metastasis-positive or metastasis-negative is obtained as the output for each image input. (**B**) The receiver operating characteristic curves of the three trained DCNN models (InceptionV3, EfficientNetB4, and DenseNet-121). The black square area has been enlarged and displayed as an inset. The areas under the curves are shown in the lower right corner of the image. (**C**) The confusion matrix of the three DCNN models on the test dataset. *DCNN* deep convolutional neural network, *MUSE* microscopy with ultraviolet surface excitation, *FFPE* formalin-fixed paraffin-embedded.

We trained three deep learning-based diagnostic prediction models (InceptionV3, EfficientNetB4, and DenseNet-121). The MUSE images of FFPE lymph nodes used for training, which were obtained from our previous study^8^, were labelled by the pathologists using HE images of serial sections. All labelled images used to train the DCNN models were of 256 × 256 patch size. To account for variations in accuracy due to the initial values of the weights, we trained each model 10 times; the models that yielded the best accuracy when using the test dataset were adopted into the pipeline for subsequent predictions. The accuracies of the three finally selected models based on the test dataset were 98.45%, 97.28%, and 97.78%, respectively, with InceptionV3 showing the best accuracy. The receiver operating characteristic curve (AUC) scores were 0.9985, 0.9982, and 0.9969, respectively, and the F1 scores were 98.48%, 97.24%, and 98.15%, respectively, as shown in Fig. 4B,C. All three models could classify lymph node metastatic cancers with very high accuracy. The threshold was set to a value that maximised the F1 score obtained using the validation dataset.

### Large-scale image classification and metastasis area mapping through the CycleGAN-DCNN pipeline

**Figure 5:**
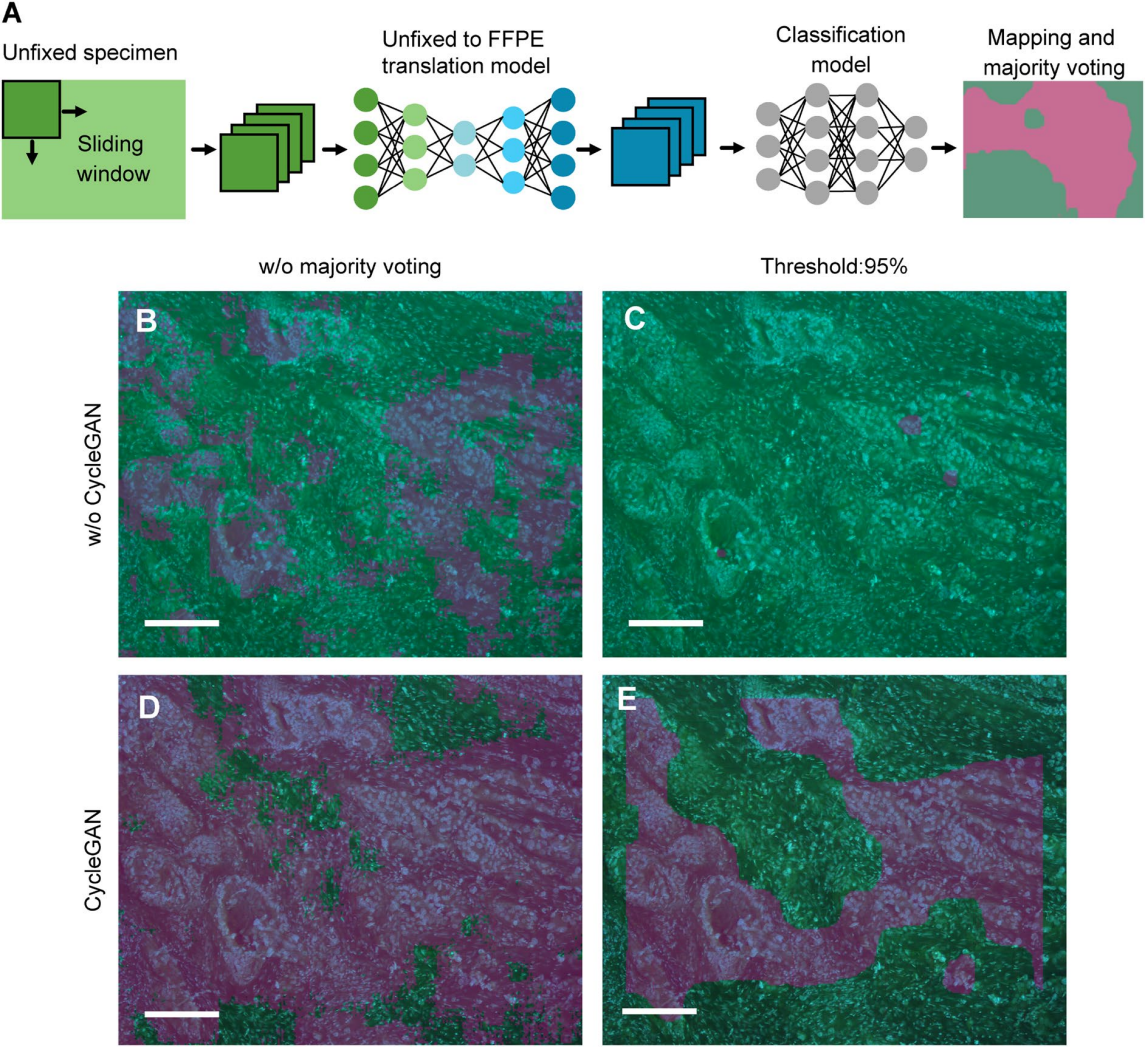
Large-scale metastasis area mapping using CycleGAN-DCNN pipeline. (**A**) Divided patches using a sliding window approach are used as the input into the trained CycleGAN, and patch-level classification is performed through the DCNN model sequentially. The majority voting processing is used as postprocessing. (**B–E**) A metastasis-positive image and its mapping results. The top (**B,C**) and bottom (**D,E**) images are original and translated images by CycleGAN, respectively. The CycleGAN-assisted preprocessing changed the contrast of the MUSE images and improved the mapping results. (**B,D**) Show images without majority voting processing, and (**C,E**) show those with majority voting processing with a 95% threshold. Scale bars: 200 µm.

As shown in Figs. 3 and 4, the image translation quality of CycleGAN was high, and the DCNN models showed high diagnostic accuracy. CycleGAN and DCNN models were then connected to create a pipeline that can predict samples being metastasis-positive or metastasis-negative based on patch images of unfixed lymph nodes. Mapping was performed using sliding window processing, and the pipeline was used to visualise metastasis-positive areas on large-scale images (Fig. 5A). We employed InceptionV3, which showed the highest prediction accuracy among the three assessed DCNNs. During the mapping process, cancerous areas were densely filled with metastasis-positive predictions, while the other areas showed a binary noise-like pattern due to sparse false positive predictions. As DCNN prediction accuracy is not 100%, such false positive predictions are thought to be inevitable. The noise pattern was removed using 95% majority voting processing for the mapping results obtained using the sliding window processing (see “Methods” section, Fig. S2). Examples of the results of each image processing are shown in Fig. 5B–E. While metastasis-positive areas could not be identified in the image before translation, the CycleGAN-assisted preprocessing in the pipeline enabled the detection of the metastasis-positive regions. The number of immunologically activated histiocytes increases in the metastatic regional lymph nodes, and they sometimes form sarcoidosis-like granulomas. These histiocytes were easily misidentified as metastasis-positive because of their large cytoplasm and low nuclear density compared to lymphocytes^8^. Large blood vessels were also misrecognised as metastasis-positive because they were similar to cancerous glandular tissue (Fig. S2B). These false positives were corrected by the 95% majority voting processing (Fig. S2D). Before majority voting processing, normal tissues surrounding cancerous tissues were widely misidentified as metastasis-positive (Fig. 5D); however, majority voting processing made it possible to recognise only the cancerous tissues (Fig. 5E).

The performance of diagnostic predictions was evaluated for the MUSE images of unfixed lymph nodes observed using a 10× objective lens. For each image and mapping results, the following rules were used for the diagnosis: even if 1 pixel of the metastasis-negative image was predicted to be positive, it was a wrong prediction, and even if 1 pixel of the metastasis-positive image was predicted to be positive, it was a correct prediction. The diagnostic prediction results for the CycleGAN-translated images, along with those obtained without the image translation, are shown in Table 1. A total of 91 images were diagnosed; 45 images from 19 cases were classified as metastasis-positive images, and 46 images from 9 cases as metastasis-negative images. The average predictive accuracy of the three DCNN models was 84.6% and 66.3% for images with and without CycleGAN translation, respectively. Additionally, all models showed a considerable increase in diagnostic accuracy when the translation with CycleGAN was introduced. AUC and F1 scores are also provided in the Supplementary Fig. S4 and Supplementary Table 1. Furthermore, see Supplementary Table 2 for results obtained when the threshold (%) for the majority voting processing was varied. The highest accuracies were achieved at all the threshold values when employing CycleGAN for preprocessing. The computational time of the three phases—CycleGAN translation, DCNN mapping, and majority voting—is shown in Supplementary Table 3.

**Table 1:** The result of large-scale image diagnostic accuracies for each of the three classification DCNN models in original unfixed specimens and upon translation by CycleGAN.

α (%)			Metastasis (−)	Metastasis (+)	Average (%)
95	Without CycleGAN	InceptionV3	38/46	23/45	67.0
		DenseNet-121	17/46	40/45	62.6
		EfficientNetB4	19/46	44/45	69.2
		Average (%)	53.6	79.3	66.3
	With CycleGAN	InceptionV3	39/46	40/45	86.8
		DenseNet-121	33/46	41/45	81.3
		EfficientNetB4	42/46	36/45	85.7
		Average (%)	82.6	86.7	84.6

The threshold of majority voting processing was set to 95%.

We visualised which area of the image before and after CycleGAN processing contributed to the prediction of the DCNN model (InceptionV3) using Grad-CAM^26^ (Fig. S3). The red areas contributed the most to the prediction, indicating that the image after CycleGAN processing could predict the diagnosis based on cancer characteristics, while those before CycleGAN processing did not recognise cancerous regions.

## Discussion

The pipeline involving the image classification model (DCNN) and image translation model (CycleGAN) proposed in this study improved the average diagnostic accuracy from 66.3 to 84.6% for MUSE images acquired using a 10× objective lens. Further improvement in accuracy can be achieved by increasing the number of patients and MUSE image data. Enhancing the quality of the image data also contributes to improved accuracy. The staining and imaging processes are performed by human operators, which can lead to variations in the data set. Mechanising and automating these processes would reduce these variations. Achieving 95% accuracy is a benchmark for operations in clinical practice. Although our study showed improved accuracy through image-translations, it’s still not sufficient for clinical use. To improve accuracy, we plan to expand our dataset and comprehensively learn various types of cancer. We hope that this will lead to more stable and accurate translations, ultimately achieving the clinical baseline accuracy.

MUSE does not require thin-sliced sections compared to conventional pathological diagnostic techniques, including HE staining protocol, and the staining process is simple and rapid. Regarding the time required for MUSE image acquisition and mapping using the pipeline, we expect that improvements in staining protocols, imaging systems, mapping algorithms, and computational speed will enable us to reduce the required time. Although the microscope used in this paper allows observation of only an area of approximately 1.2 × 1.0 mm^2^ at a time, there is room for more extensive imaging with improved optics in the future. It is also possible to reduce the time required for staining and pipeline model calculations.

The combination of MUSE and DCNN offers the following advantages. First, the combined method can reduce the burden on pathologists. As the incidence of cancer increases, the number of diagnoses per pathologist would also increase^27^. Highly accurate rapid intraoperative diagnosis systems combining rapid imaging technology and automatic AI-assisted diagnosis will increase their importance in pathology. Second, variations in diagnostic performance among pathologists can also be minimised. Third, our method can provide expert-level diagnosis where pathology resources are scarce.

A sufficient number of images is required for the training of classification models, such as DCNN; however, it is difficult to collect a large number of MUSE images of unfixed specimens and to cover all pathological findings at a single facility. Notably, the method proposed in our study does not require a large number of images of unfixed specimens for training the DCNNs. Our method only needs a relatively small number of images of unfixed specimens for CycleGAN training and a large amount of image data can be generated from previous FFPE samples stored at the facility for classification model training. By using FFPE samples to prepare large amounts of image datasets, including those for rare diseases, it would become possible to create image classification models that can predict diagnosis with high accuracy for a variety of diseases. Without the CycleGAN image translation process, we will need to devote a significant effort to creating clinical image datasets of rare diseases from unfixed specimens for accurate diagnostic prediction.

Our results show that CycleGAN-based image translation improved diagnostic performance even for different specimens prepared using different processes. CycleGAN has the potential to handle not only the unfixed to FFPE MUSE image translation but also other intraoperative diagnostic imaging modalities, such as two-photon excited autofluorescence (TPEF), second harmonic generation (SHG), third harmonic generation (THG), and stimulated Raman scattering (SRS) microscopy, and would reduce the difficulties associated with clinical image data acquisition. These nonlinear optical microscopies do not require thin sectioning and staining with conventional methods. Additionally, deep learning-based image classification models enable the diagnosis of images obtained by a new imaging method that general pathologists cannot diagnose^28–31^. Among these new imaging modalities, MUSE imaging offers a cost-effective, accessible alternative to complex nonlinear optical methods. It uses a low-cost LED light source and is as easy to use as standard fluorescence microscopy, making it practical and suitable for clinical applications.

A chief limitation of our study is that we only evaluated lymph node metastasis in gastric cancer. The robustness of the model should be assessed through external validation. To address this, the dataset will be expanded to include other cancer samples obtained from several facilities in the future. A secondary limitation is that the edge of the images is not mapped after the majority voting process. This problem will be solved in the future by replacing the image classification task using DCNN and the majority voting process with segmentation models. As the entire dataset was used to train CycleGAN in this study, further research is warranted to validate the generalisation of its performance for clinical settings.

## Conclusion

MUSE microscopy was applied for pathological diagnosis of lymph node metastasis in gastric cancer. The modality translation using CycleGAN improved the pathological diagnosis of non-thin-sliced surface images using DCNN model trained with FFPE thin-sliced images. Our method enables highly accurate diagnosis even with a small number of images of unfixed samples when combined with existing images of FFPE samples and can be potentially applied to rapid intraoperative diagnostic images acquired using various microscopes.

### Supplementary Information

Supplementary Information.

## Data Availability

The datasets generated and analysed during the current study are not publicly available due to privacy concerns of the patients, but are available from the corresponding authors on reasonable request.

## Abbreviations

DUV: Deep-ultraviolet
FFPE: Formalin-fixed paraffin-embedded
DCNN: Deep convolutional neural network
MUSE: Microscopy with ultraviolet surface excitation
TPEF: Two-photon excited autofluorescence
SHG: Second harmonic generation
THG: Third harmonic generation
SRS: Stimulated Raman scattering
