# Supplementary material for: Deep UV-excited fluorescence microscopy installed with CycleGAN-assisted image translation enhances precise detection of lymph node metastasis towards rapid intraoperative diagnosis
Source: Sci Rep. 2023 Dec 4;13:21363. doi: 10.1038/s41598-023-48319-7 (PMC10696085; doi:10.1038/s41598-023-48319-7)
Supplement: Supplementary file 1 — Supplementary Information. [file 41598_2023_48319_MOESM1_ESM.docx]

**Deep UV-excited fluorescence microscopy installed with CycleGAN-assisted image translation enhances precise detection of lymph node metastasis towards rapid intraoperative diagnosis**

Junya Sato^†, 1, 4, 5^, Tatsuya Matsumoto^†, 2^, Ryuta Nakao^†, 2^, Hideo Tanaka^2^, Hajime Nagahara^1, 3^, Hirohiko Niioka*^, 1^, Tetsuro Takamatsu*^, 2, 6^

^†^ These authors equally contributed

^1^ Graduate School of Information Science and Technology, Osaka University, 1-5, Yamadaoka, Suita, Osaka 565-0871, Japan

^2^ Department of Pathology and Cell Regulation, Kyoto Prefectural University of Medicine, 465 Kajiicho, Kawaramachi-Hirokoji, Kamigyo-ku, Kyoto, 602-8566, Japan

^3^ Institute for Datability Science, Osaka University, Yamadaoka, 2-8 Suita, 565-0871, Japan

^4^ Department of Radiology, Osaka University Graduate School of Medicine, 2-2, Yamadaoka, Suita, Osaka 565-0871, Japan

^5^ Department of Artificial Intelligence Diagnostic Radiology, Osaka University Graduate School of Medicine, 2-2, Yamadaoka, Suita, Osaka, 565-0871, Japan

^6^ Department of Medical Photonics, Kyoto Prefectural University of Medicine, 465 Kajiicho, Kawaramachi-Hirokoji, Kamigyo-ku, Kyoto, 602-8566, Japan

***Corresponding authors:**

Hirohiko Niioka, PhD

Graduate School of Information Science and Technology, Osaka University

2-8 Yamadaoka, Suita, Osaka, 565-0871, Japan

Phone: +81-6-6879-4141

Fax: +81-6-6879-4141

E-mail: [niioka@ist.osaka-u.ac.jp](mailto:niioka@ist.osaka-u.ac.jp)

Tetsuro Takamatsu, MD, PhD

Department of Medical Photonics, Kyoto Prefectural University of Medicine, 465 Kajiicho, Kawaramachi-Hirokoji, Kamigyo-ku, Kyoto, 602-8566, Japan

Phone: +81-75-251-5538

Fax: +81-75-251-5353

E-mail: [ttakam@koto.kpu-m.ac.jp](mailto:ttakam@koto.kpu-m.ac.jp)


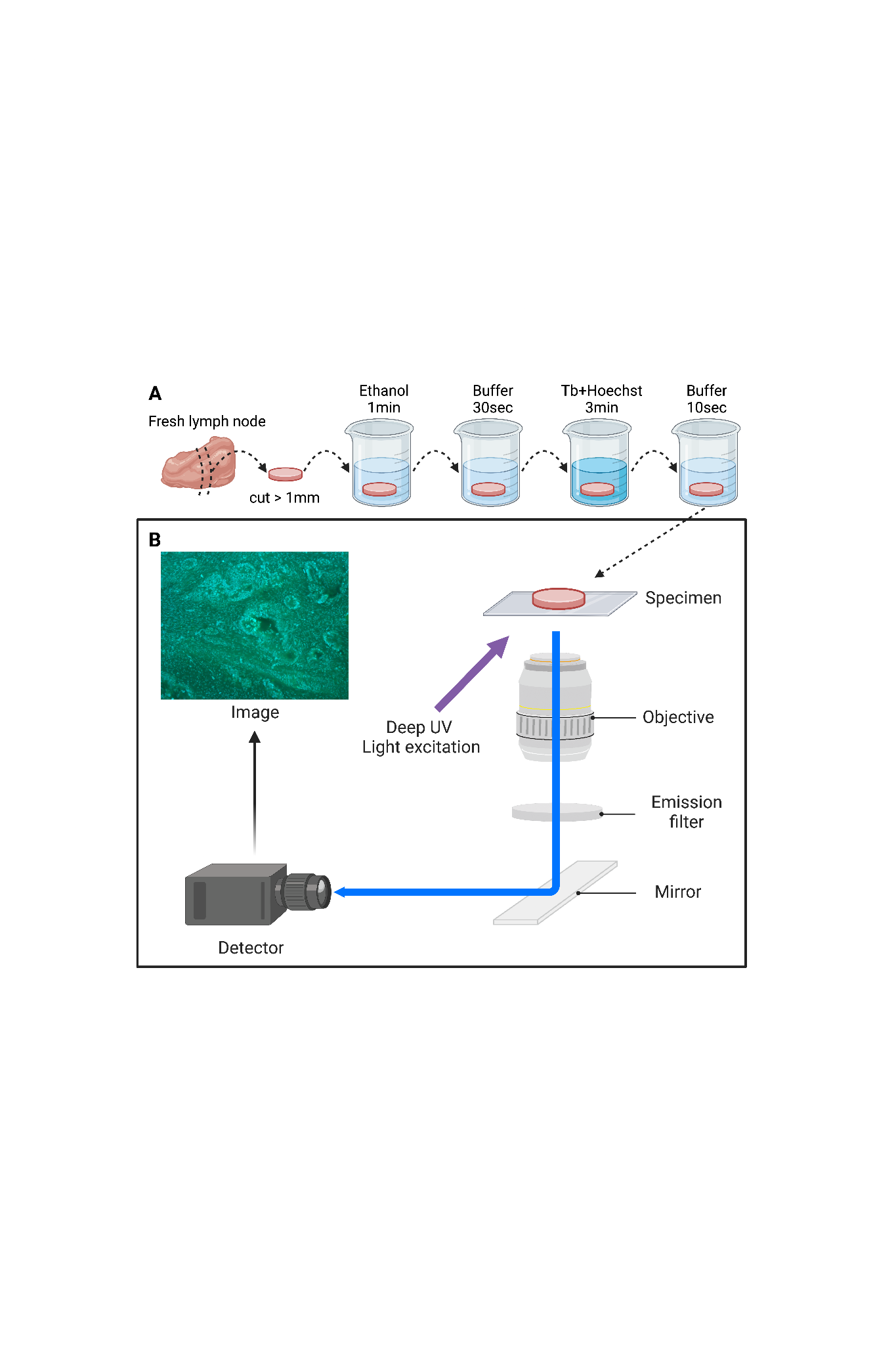


**Figure S1**. A schematic representation of the MUSE image acquisition protocol. (A) A fresh lymph node obtained from surgery is sliced and stained with several solutions, such as Tb^3+^ and Hoechst solution. The cytoplasm and nucleus are stained using Tb^3+^ and Hoechst, respectively. (B) The specimen surface is illuminated using DUV light from an oblique downward angle. Fluorescence in the visible light range emitted from the specimen is collimated with the objective lens and recorded using a CMOS camera.

**
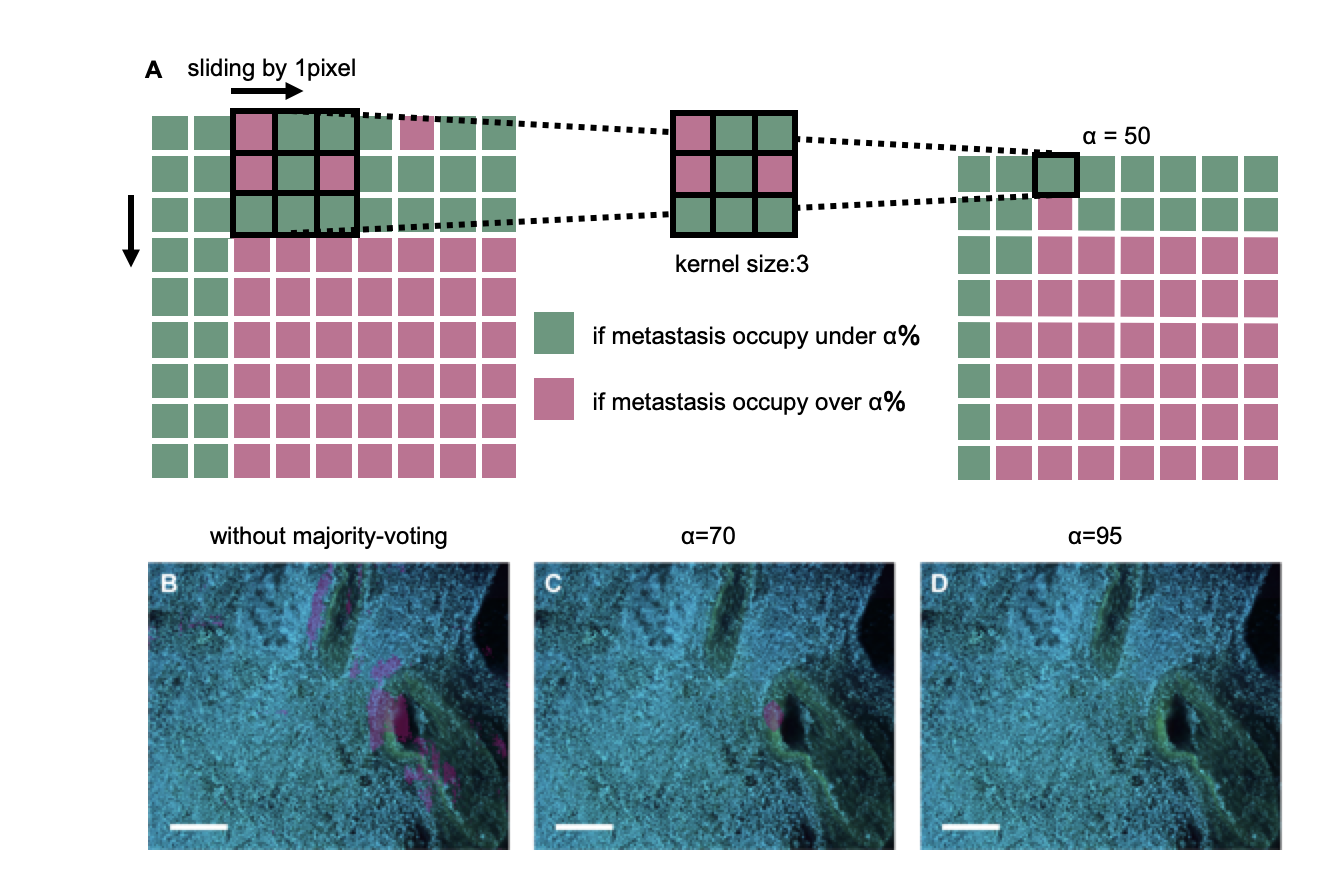
**

**Figure S2**. (A) Schematic diagram of the majority voting process. Green and pink rectangles indicate a pixel predicted as metastasis-negative and metastasis-positive, respectively. The kernel represents the region where majority voting is performed, and this region scans the entire image with a step size of 1 pixel. As an example, there are 3 metastasis-positive pixels and 6 metastasis-negative pixels in the 3 × 3 kernel, and the output result is metastasis-negative when the majority voting threshold α is set to 50%; the actual kernel size applied in this study was 26 × 26. (B–D) A metastasis-negative image and its mapped results by CycleGAN. (B) shows the image without majority voting processing, and (C) and (D) show those with majority voting processing with thresholds set to 70% and 95%, respectively. Scale bars: 200 µm.


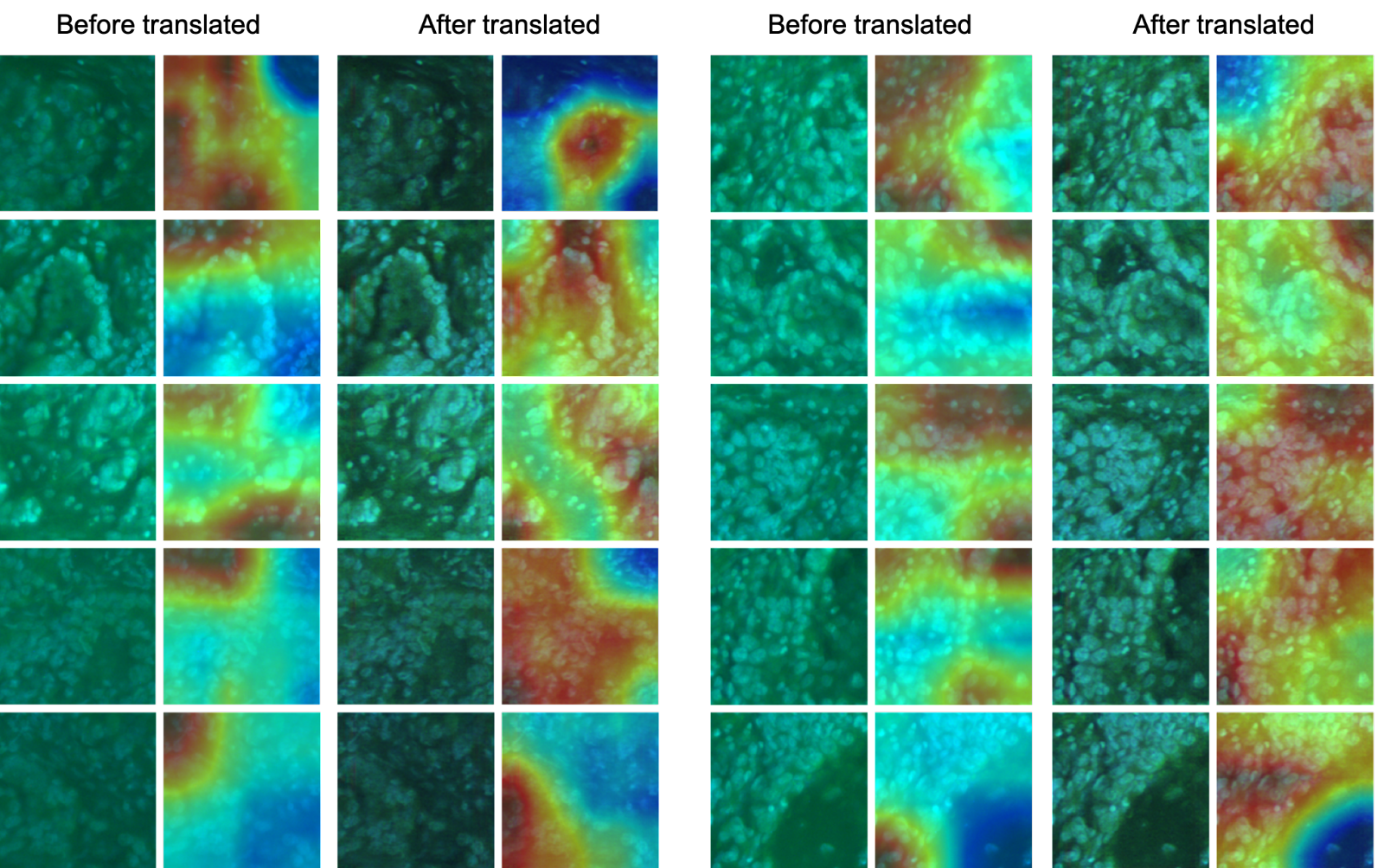


**Figure S3**. Grad-CAM visualisations before and after CycleGAN-assisted translation. The results for 10 patch images are shown. Grad-CAM activation maps are shown on the right side of each patch image. In the translated image, the region of interest in the DCNN model, shown in red, overlaps the metastatic cancer region.


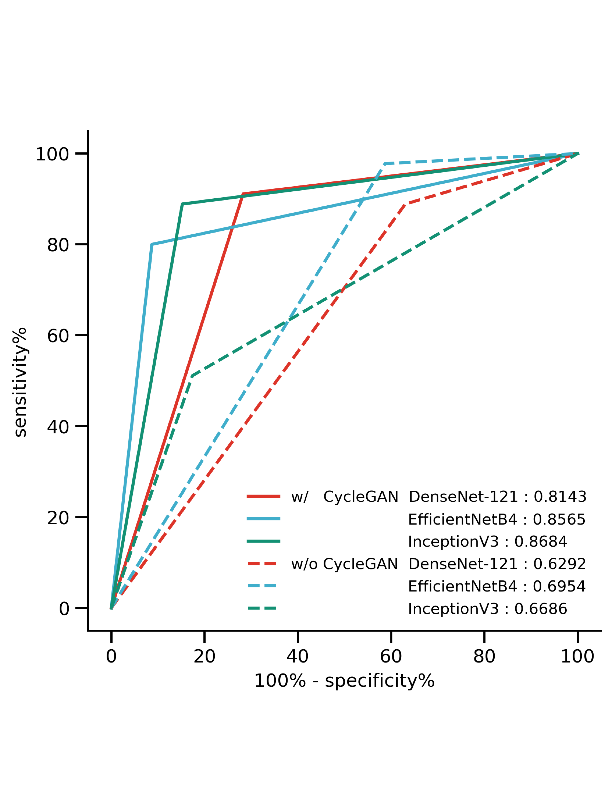


**Figure S4**. The receiver operating characteristic curves of the three trained DCNN models (InceptionV3, EfficientNetB4, and DenseNet-121) on large-scale images. Note that the solid lines represent the results without CycleGAN, and the dotted lines represent the results with CycleGAN The areas under the curves are shown in the lower right corner of the image.

**Supplementary Table 1.** The receiver operating characteristic curve (AUC) and F1 scores for large-scale images of DCNN models with and without CycleGAN.

|  |  | F1 | AUC |
| --- | --- | --- | --- |
| without  CycleGAN | InceptionV3 | 60.5 | 66.9 |
|  | DenseNet-121 | 70.2 | 62.9 |
|  | EfficientNetB4 | 75.9 | 69.5 |
| with CycleGAN | InceptionV3 | 87.0 | 86.8 |
|  | DenseNet-121 | 82.8 | 81.4 |
|  | EfficientNetB4 | 84.7 | 85.7 |

**Supplementary Table 2.** The result of large-scale image diagnostic accuracies with varied thresholds of 90%, 80%, and 70% in the majority voting processing.

| α |  |  | Metastasis(-) | Metastasis(+) | Avarage(%) |
| --- | --- | --- | --- | --- | --- |
| 90 | w/o  CycleGAN | InceptionV3 | 37/46 | 28/45 | 71.4 |
|  |  | Densenet121 | 16/46 | 41/45 | 62.6 |
|  |  | EfficientNetB4 | 16/46 | 44/45 | 65.9 |
|  |  | Average(%) | 50.0 | 83.7 | 66.7 |
|  | CycleGAN | InceptionV3 | 37/46 | 43/45 | 87.9 |
|  |  | Densenet121 | 29/46 | 42/45 | 78.0 |
|  |  | EfficientNetB4 | 40/46 | 37/45 | 84.6 |
|  |  | Average(%) | 76.8 | 90.4 | 83.5 |
| 80 | w/o  CycleGAN | InceptionV3 | 37/46 | 31/45 | 74.7 |
|  |  | Densenet121 | 14/46 | 43/45 | 62.6 |
|  |  | EfficientNetB4 | 16/46 | 44/45 | 65.9 |
|  |  | Average(%) | 48.6 | 87.4 | 67.8 |
|  | CycleGAN | InceptionV3 | 36/46 | 45/45 | 89.0 |
|  |  | Densenet121 | 24/46 | 45/45 | 75.8 |
|  |  | EfficientNetB4 | 34/46 | 38/45 | 79.1 |
|  |  | Average(%) | 68.1 | 94.8 | 81.3 |
| 70 | w/o  CycleGAN | InceptionV3 | 36/46 | 34/45 | 76.9 |
|  |  | Densenet121 | 12/46 | 43/45 | 60.4 |
|  |  | EfficientNetB4 | 12/46 | 45/45 | 62.6 |
|  |  | Average(%) | 43.5 | 90.4 | 66.7 |
|  | CycleGAN | InceptionV3 | 35/46 | 45/45 | 87.9 |
|  |  | Densenet121 | 18/46 | 45/45 | 69.2 |
|  |  | EfficientNetB4 | 31/46 | 40/45 | 78.0 |
|  |  | Average(%) | 60.9 | 96.3 | 78.4 |

The results of the three prediction models were computed on both original images of unfixed specimens and those translated by CycleGAN.

**Supplementary Table 3**. Computational time for large-scale image and patch image in CycleGAN translation, DCNN mapping, and majority voting.

|  | Time per large-scale  image (s) | Time per patch (ms) |
| --- | --- | --- |
| CycleGAN translation | 75.1 | 1.42 |
| DCNN mapping | 61.0 | 1.15 |
| Majority voting | 0.94 |  |
| Total time | 137 |  |
